# Multiscale Damage and Failure Behavior of Drainage Asphalt Mixture Under Multifactor

**DOI:** 10.3390/ma18214924

**Published:** 2025-10-28

**Authors:** Xiong Tao, Tao Bai, Jianwei Fan, Haiwei Shen, Hao Cheng

**Affiliations:** 1 Hubei Provincial Expressway Industrial Development Co., Ltd., Wuhan 430000, China; tx900702@163.com; 2 School of Materials Science and Engineering, Wuhan Institute of Technology, Wuhan 430205, China; yixiaotian0113@126.com; 3 School of Transportation, Southeast University, Nanjing 211189, China; fanjianwei@seu.edu.cn (J.F.); 15834511949@163.com (H.C.)

**Keywords:** damage development, failure development, fatigue life fitting, multifactor, drainage asphalt mixture, interface molecular simulation

## Abstract

Macroscopic fatigue tests, mesoscopic finite element simulations, and microscopic molecular dynamics simulations were composed to study the damage and failure of drainage asphalt mixtures in multiscale. The applicability of the fatigue models fit by strain, stress, and the linear fitting slope of the indirect tensile modulus curves were compared. The mesoscopic damage and failure distribution and evolution characteristics were studied, considering the single or coupling effect of traffic loading, hydrodynamic pressure, mortar aging, and interfacial attenuation. The microscopic molecular mechanism of the interface adhesion failure between the aggregate and mortar under water-containing conditions was analyzed. Results show that the fatigue model based on the linear fitting slopes of the indirect tensile modulus curves has significant applicability for drainage asphalt mixtures with different void rates and gradations. The damage and failure have an obvious leap development when traffic loading increases from 0.7 MPa to 0.8 MPa. The hydrodynamic pressure significantly increases the stress of the mortar around the voids and close to the aggregate, promoting damage development and crack extension, especially when it is greater than 0.3 MPa. With the aging deepening of the mortar, the increase rate of the damage degree gradually decreases from the top to the bottom of the mixture. With the development of interfacial attenuation, the damage and failure of interfaces continue increasing, while that of the mortar increases first and then decreases, which is related to the loading concentration in the interface and the stress decrease in the mortar. Under the coupling effects, whether the cracks mainly generate in the mortar or interface depends on their damage degrees, thus causing the stripping of the aggregate wrapped or not wrapped by the mortar, respectively. The van del Waals force is the main molecular effect of interface adhesion, and both acidic and alkaline aggregate components significantly tend to form hydrogen bonds with water rather than asphalt, thus attenuating the interface adhesion.

## 1. Introduction

During the operation phase, the high viscosity asphalt mortar in the drainage asphalt pavement continuously ages, and the mechanical properties of the interface between the mortar and the aggregate continuously deteriorate. Under the coupling effect of external factors such as top traffic loading, internal hydrodynamic pressure, and internal factors such as mortar aging and interfacial attenuation, the damage and failure in drainage asphalt mixture are exacerbated [1,2,3]. The traffic load mainly depends on the axle load of the vehicles, and the magnitude of the hydrodynamic pressure inside the voids can be calculated using analytical methods such as the effective stress principle or Darcy’s law, while the more common method is the fluid structure coupling numerical simulation. The value of hydrodynamic pressure is related to multiple factors, such as traffic loading, structural thickness, material type, and void water saturation degree [4,5,6].

The fatigue life measured by laboratory tests is a macroscopic characterization of the damage and failure development of asphalt mixtures. Mapping models between fatigue life and stress or strain is often constructed, but it is only applicable to the same type of asphalt mixture [7,8]. In reference [7], a fatigue life model was proposed based on the modulus attenuation curve of specimens in an indirect tensile test, which was applicable to mixtures with different void ratios and gradations. However, its applicability to drainage asphalt mixtures has not been verified. In laboratory experiments, traffic loads are relatively easy to control, and aged mixture specimens can be prepared under multiple conditions such as thermal oxygen, ultraviolet radiation, and moisture [9,10]. For the hydrodynamic pressure in the void structure, the common method is to pre-immerse or freeze–thaw the specimens before loading or apply the load under immersion conditions, but the magnitude of the hydrodynamic pressure during loading cannot be controlled easily [11,12]. Researchers have designed a hydrodynamic flushing test instrument to simulate the flushing effect of water in voids by adjusting the internal air pressure of the instrument to apply different hydrodynamic pressures, but it cannot achieve the coupled application of traffic loads [13]. In addition, it is difficult to quantify the mechanical properties such as the adhesion at the interface of the specimens. Therefore, numerical simulation is an effective method for quantifying the coupling effects of the internal and external factors mentioned above. By constructing finite element or discrete element models of drainage asphalt pavements, different aging states can be characterized by adjusting material mechanic parameters, and the coupling effect of traffic loads and hydrodynamic pressure can be applied [14,15].

The interface adhesion requirements between the high viscosity asphalt mortar and the aggregate in the drainage asphalt mixture are much higher than those of ordinary asphalt mixtures. The closer the elastic–plastic index of the interface is to the mortar, the less likely it is to have stripping diseases. However, in current studies, interfacial attenuation is mostly discussed as a phenomenon in the evolution of damage and failure, while the influence of the initial state of the interface on the subsequent damage and failure development of the mixture is mostly ignored [16,17,18,19]. Therefore, it is necessary to consider the initial interface attenuation state as a factor in the study of damage and failure behavior. In addition, when the drainage asphalt mixture is severely damaged, moisture diffuses into the interface, exacerbating the attenuation of the interface adhesion. This effect is difficult to quantitatively characterize in laboratory experiments, but molecular dynamics simulations can be applied to reveal the mechanism of the above interface action at the molecular microscopic level [20,21].

In this paper, macroscopic fatigue tests, mesoscopic finite element simulations, and microscopic molecular dynamics simulations were composed to study the damage and failure of drainage asphalt mixtures in multiscale. A fatigue model construction method applicable to drainage asphalt mixtures with different void ratios and gradations was put forward to describe the damage and failure behavior in the macro scale. The mesoscopic damage and failure distribution and evolution characteristics were studied, considering the single or coupling effect of internal and external factors, including traffic loading, hydrodynamic pressure, mortar aging, and interfacial attenuation. The microscopic molecular mechanism of the interface adhesion failure between aggregate and mortar under water-containing conditions was analyzed.

## 2. Testing, Modeling, and Validation

### 2.1. Materials and Specimen Preparation

The indicators of high viscosity asphalt, basalt coarse and fine aggregates, and limestone mineral powder are shown in Table 1. The gradation curves of the prepared PAC13, PAC16, and PAC20 mixtures are shown in Table 2, with the asphalt contents of 5.0%, 5.3%, and 5.1%, respectively.

According to the Chinese specification of Standard Test Methods of Bitumen and Bituminous Mixtures for Highway Engineering (JTG E20-2011) [22], cylindrical specimens for indirect tensile testing were prepared by gyratory compaction. The height of specimens is 60 mm, and the radius is 50 mm. Two levels of void rate, i.e., 18% and 22%, were set for various types of drainage asphalt mixture specimens by adjusting the number of compaction cycles.

### 2.2. Indirect Tensile Test

The indirect tensile test was conducted according to the European specification of Bituminous Mixtures–Test Methods for Hot Mix Asphalt: Resistance to Fatigue [22]. The temperature was 20 °C, and the stress control method was applied. The applied horizontal tensile stress σ is shown in Table 3, and Equation (1) was applied to set the corresponding half sine peak load P. In the equation, R is the radius and H is the height of the specimen.

(1) P=πσRH

Each loading cycle is 0.5 s, with a half sine load applied for 0.1 s. The number of load cycles corresponding to the tensile failure of the specimen is the fatigue life $N_{f}$. Three parallel specimens were set for each type of mixture to test and calculate their average fatigue life.

The initial tensile strain ε of the specimen can be calculated by Equation (2), in units of micro strain [23]. In the equation, ΔD represents the difference between the total deformation of the specimen during the 100th cycle and the elastic deformation of the specimen during the 60th cycle of indirect tensile loading.

(2) $$\varepsilon =\frac{1.05\Delta D}{R}$$

The indirect tensile modulus E of the specimen was calculated according to Equation (3), where D was the peak horizontal elastic deformation of the specimen during a single loading, and *μ* was Poisson’s ratio [24].

(3) $$E=\frac{P(0.27+\mu )}{DH}$$

### 2.3. Mesoscopic Model Construction and Parameter Calibration

The Abaqus software (version number 6.10) was applied, and based on the random aggregate placement algorithm, coarse aggregate with a particle size larger than 2.36 mm were placed according to the gradation, and the fine aggregate, mineral powder, and high viscosity asphalt were combined to form the asphalt mortar. The voids were generated, adopting the quartet-structure generation-set method, and the mutual interference between aggregates and voids was eliminated, so as to form a mesoscopic model of drainage asphalt mixture [25,26]. Due to the difference between the 3D and 2D void rates, based on the computed tomography (CT) scan results of PAC13 specimens, the void rate of the Mesoscopic model was set to be 11.5%. The 2D model of the PAC13 asphalt mixture has a dimension of 50 mm × 50 mm, and it has a fixed bottom boundary and free boundaries on both the left and right sides.

By conducting trial calculations to determine the grid size, significant differences were observed in the calculation results when the grid accuracy varied between 0.5 mm and 2 mm, indicating a significant effect of grid size. When the grid accuracy varies between 0.1 mm and 0.5 mm, the calculation results of the damage cloud map are basically equivalent, indicating that the size effect of the grid has been basically eliminated and stabilized. Taking into account factors such as the size, calculation accuracy, and efficiency of the mesoscopic model, a grid accuracy of 0.5 mm was selected. Due to the presence of some narrow and elongated mesh areas in the mesoscopic model, a mesh partitioning mode using triangular elements and free sweep was adopted to ensure the mesh quality. The dynamic explicit calculation was set in the analysis step to improve the convergence of the model calculation. Continuing to reduce the grid size results in a difference of less than 2% in the subsequent simulation results.

**Figure 1:**
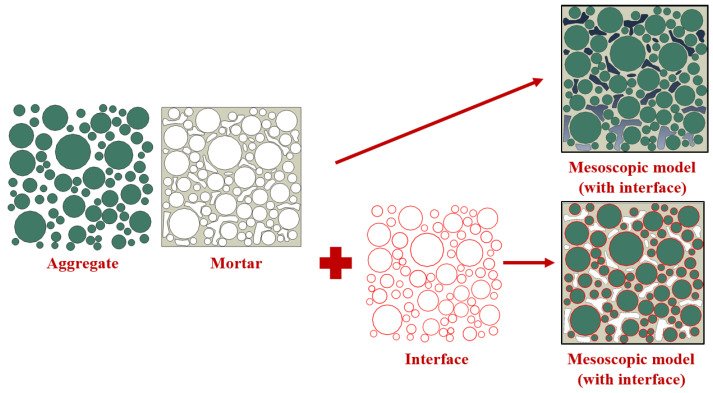
Mesoscopic models of drainage asphalt mixture with or without independent interface.

The original aggregate particles were divided from the inside out into two parts, that is, aggregate and interface, so as to construct the Mesoscopic models with aggregate–mortar interfaces. Considering the distribution of free and structural asphalt, the interface thickness of 0.1 mm was decided, and a four-phase composite model including the aggregate, mortar, interface, and voids was obtained. The Mesoscopic models with and without the interface are shown in Figure 1.

The elastic constitutive parameters of high viscosity asphalt mortar were selected from the data at 20 °C and 10 Hz loading, with a modulus of 3500 MPa and Poisson’s ratio of 0.35 [27,28]. The tensile test was conducted to test the true stress–true strain curves of asphalt mortar, so as to calculate the plastic constitutive parameters. The Ductile damage model was applied, and the fracture strain was the plastic strain value corresponding to the stress peak on the stress–strain curve. The smaller the value, the earlier the material begins to suffer damage. Fracture energy is determined by integrating the descending phase of the curve, and the larger the value, the longer the damage development stage of the material and the better its ductility [29,30].

**Figure 2:**
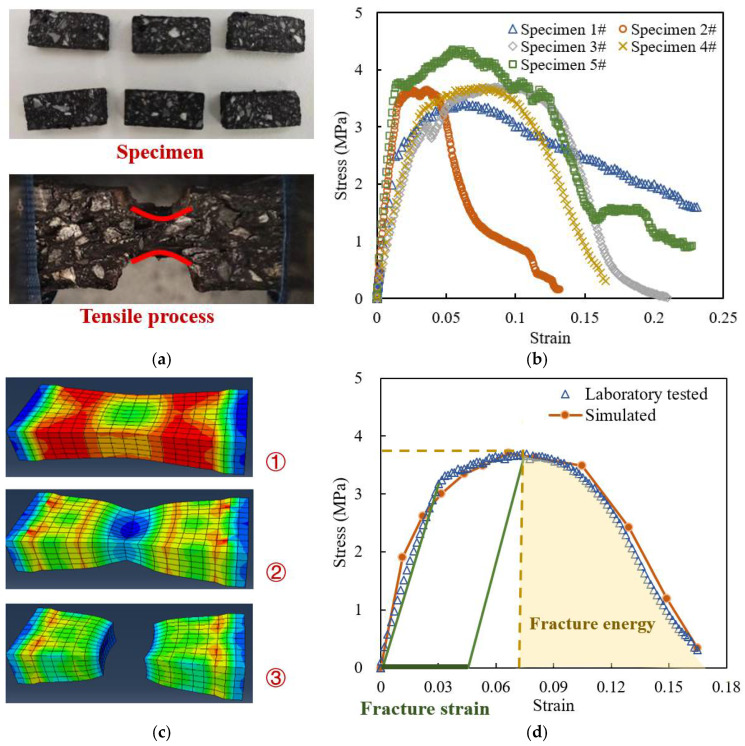
Calibration and verification of Mesoscopic parameters of asphalt mortar: (**a**) laboratory test; (**b**) stress–strain curves obtained by laboratory tests; (**c**) virtual simulated test; (**d**) comparison of stress–strain curves between laboratory tested and simulated.

PAC13 is one of the most commonly used drainage asphalt upper layer materials, while PAC16 and PAC20 are only used as the middle surface layer materials in a few double-layer drainage pavements, with a relatively small application range [31,32]. Therefore, in the tensile test of asphalt mortar, basalt fine aggregate and high viscosity asphalt were applied, and PAC13 gradation and asphalt content in Section 2.1 were selected to prepare asphalt mortar specimens. The size of the tensile test specimen is 20 mm × 10 mm × 50 mm, and the loading rate is 0.1 mm/s. Double-sided notches were pre-set in the middle of the specimen to ensure cracking in the middle, as shown in Figure 2a. Compared with matrix asphalt mortar, the tensile process of high viscosity modified asphalt mortar is very close to that of metal at room temperature, and it also shows a “necking” phenomenon.

The tensile test results of the five specimens are shown in Figure 2b. It can be seen that the development trends of various curves during the tensile test are roughly similar, and each specimen undergoes three stages of elasticity, plasticity, and damage. The first is the elastic stage, during which the stress–strain of the material exhibits a linear relationship. The second is the plastic development stage, during which the strain increases rapidly, but the stress increases slowly. When the stress reaches its highest value, it enters the damage stage, during which the stress begins to decrease with the development of strain, indicating that the material’s damage begins to accumulate. Finally, the stress drops until complete failure of the material. Therefore, the tensile cracking phenomenon and the strain–stress curves both show ductile damage characteristic like metal, so the Ductile Damage Model in Abaqus software was applied [33,34,35].

In Figure 2b, the stress–strain curve of specimen 4# has good continuity, and it is close to the mean of the results of other specimens. Therefore, the curve of specimen 4# was selected to calculate the plastic constitutive parameters of high viscosity asphalt mortar. It can be calculated that the fracture strain is 0.0343 and the fracture energy is 5.97 kJ/m^2^.

The aforementioned elastic–plastic parameters were applied, and a finite element virtual tensile test was conducted, as shown in Figure 2c. In the virtual test, the specimen size and loading parameters were the same as the actual ones. The elements at the center of the specimen section were selected to calculate the true axial strain and stress. As shown in Figure 2d, the simulated and laboratory tested stress–strain curves are relatively close in the elastic, plastic, and damage stages. The relative difference (RD) of the two curves is calculated by Equation (4), where $S_{s}(S_{n})$ is the strain ($S_{n}$)–stress ($S_{s}$) fold line obtained by linear interpolation of the simulated values, ${S_{s}}^{\prime }(S_{n})$ is the strain ($S_{n}$)–stress (${S_{s}}^{\prime }$) fold line obtained by linear interpolation of the laboratory tested values, and $S_{n,max}$ is the maximum strain value. A lower RD value means lower difference between the simulated and laboratory tested curves.

(4) $$RD=\frac{\int_{0}^{S_{n,max}}\left|S_{s}\left(S_{n}\right)-{S_{s}}^{\prime }(S_{n})\right|d(S_{n})}{\int_{0}^{S_{n,max}}S_{s}^{\prime }\left(S_{n}\right)d(S_{n})}$$

The calculated RD value is 4.96%, which indicates that the average difference between the two curves is less than 5%. Although the 5% difference is acceptable, the point where the linear behavior of the elasticity zone of the curve obtained from the molecular simulation ends does not correspond to the point where the same linear behavior of the experimental curve ends. The above curve difference is related to the difference between the morphological and constitutive parameters of the microscopic model and the actual specimen, and it will be further discussed in our future studies.

Furthermore, according to Section T0713 in the Chinese specification of Standard Test Methods of Bitumen and Bituminous Mixtures for Highway Engineering (JTG E20-2011) [22], the uniaxial compression creep test was conducted, and specimens were prepared according to the gradation and asphalt content of PAC13 mixture in Table 1.

A constant load of 0.7 MPa was applied, and the strain–time curve during the loading process was recorded, with three parallel specimens. In addition, finite element simulation of uniaxial compression creep test was conducted, and the concentrated force was applied through the indenter on the top of the model.

**Figure 3:**
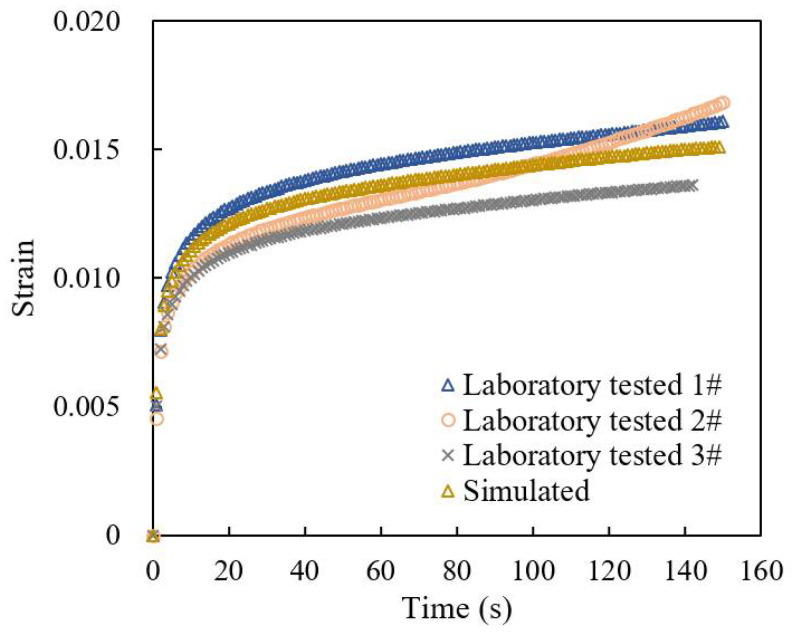
Strain–time curves during laboratory uniaxial compression creep test and simulation.

The displacement of the indenter in the simulation was output to calculate the model strain, and it was compared with the measured strain curve, as shown in Figure 3. The creep strain pattern of the simulated strain curve is similar in trend to the three laboratory tested curves, with a strain value error within 5%. In summary, the constructed microscale finite element model can be applied to simulate the elastic–plastic damage behavior of the drainage asphalt mixture under load.

**Figure 4:**
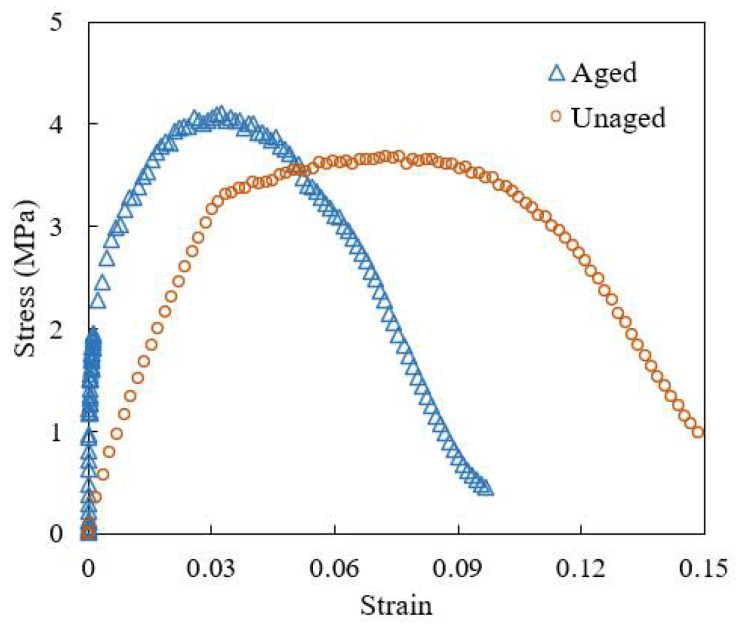
Stress–strain curve of asphalt mortar before and after aging.

According to the accelerated aging method in the T0734 section of the Chinese standard of JTG E20-2011 [22], the high viscosity asphalt mortar prepared in Section 2.3 was placed in an 85 °C oven for 1 day, so as to prepare the aged specimens. As in Section 2.3, the tensile curves of the aged and unaged specimens were tested, as shown in Figure 4. The calculated fracture strain after aging was 0.0242, and the fracture energy was 4.715 kJ/m^2^. On this basis, five sets of parameters to were determined to characterize different aging stages of mortar, as shown in Table 4.

### 2.4. Traffic Loading and Hydrodynamic Pressure Application

**Figure 5:**
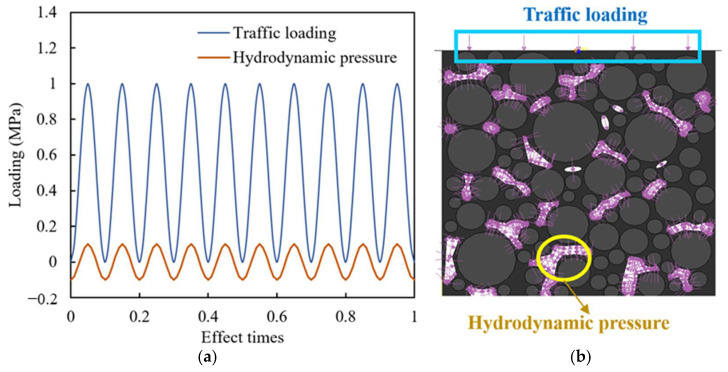
Hydrodynamic pressure and traffic loading: (**a**) synchronous phase combination; (**b**) diagram of load application.

When there is a large amount of precipitation, the voids in the mixture are completely filled with water. If a vehicle load passes through at this time, the impact of the dynamic water pressure on the mixture is the strongest. The dynamic water pressure at this time can be simulated using a simply harmonic wave. Therefore, considering this extreme situation, a 10 Hz semi vector function load was applied at the top of the model to simulate the traffic load. When the vehicle comes in and leaves, it squeezes water into the voids or draws water from it, forming positive and negative hydrodynamic pressure, respectively. Affected by factors such as vehicle speed and pavement moisture content, the internal hydrodynamic pressure of the asphalt pavement ranges from 0.1 MPa to 0.5 MPa [5,36,37]. The hydrodynamic pressure is in phase with the traffic load, that is, when the traffic load reaches its maximum and minimum values, the hydrodynamic pressure inside the voids also reaches the maximum positive and negative values, respectively, as shown in Figure 5a. The direction of hydrodynamic pressure application is normal to the surface of the void, as shown in Figure 5b. To figure out the coupling effect of traffic loading and hydrodynamic pressure, the hydrodynamic pressure and traffic load were first applied together 1500 times, and then the traffic load was applied separately 500 times.

### 2.5. Microscopic Molecular Interface Model

**Figure 6:**
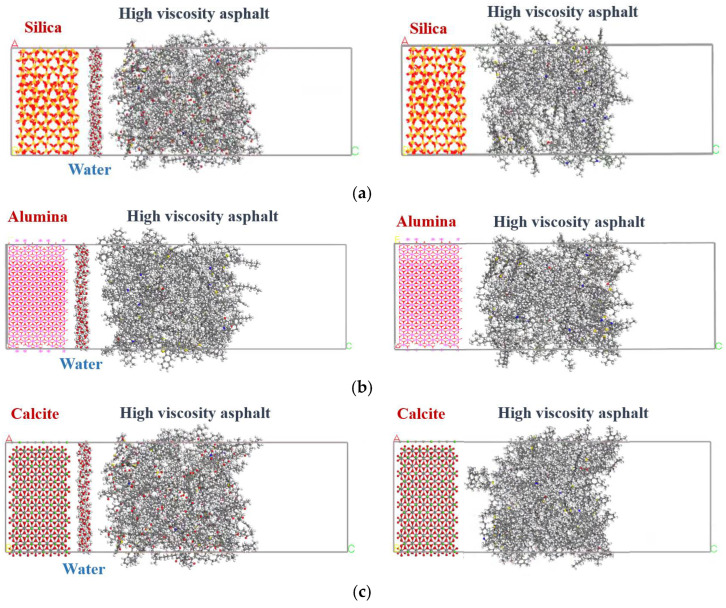
Interface molecular models with or without water: (**a**) silica; (**b**) alumina; (**c**) calcite.

When there is no obvious destruction in the drainage asphalt mixture, water only exists in its void structure. However, when it is severely destructed, water enters the interface between the aggregate and mortar through cracks in the mortar, exacerbating the attenuation of interfacial adhesion. To investigate the molecular mechanism of the water influence on the interface, interface molecular models with or without water were both constructed, as shown in Figure 6.

On the left side of the interface model, crystal cells of silica, alumina, or calcite were selected and placed. Silica and alumina are main components of common aggregates such as granite, basalt, and diabase, while calcite is the main component of limestone. On the right side of the interface model, the high viscosity asphalt models were placed, which is composed of asphalt, styrene–butadiene–styrene (SBS), and light oil molecules. The 12-component asphalt model was selected, and an n-octane molecule model was applied to represent the light oil. The chain SBS molecular fragments were built by the sequential polymerization of six styrene, three 1,3-butadiene, and six styrene molecules. According to the actual measured results of a certain high viscosity agent, the number ratio between SBS and light oil molecules was set to be 1:3, and the sum of their molecular masses accounted for about 12% of the total mass of high viscosity asphalt [13,38,39,40].

The Material Studio software (version number 4.0) was applied, and all of the calculations were conducted in the vacuum environment. After the molecule models were placed in the interface model crystals, the models were, firstly, geometrically optimized under the COMPASSII force field with 5000 iterations to eliminate the unreasonable structure of the system. Then, five cycles of the annealing process were conducted under the NVT (i.e., the number of atoms N, volume V, and temperature T of the model remained unchanged) ensemble at the temperature range from 598 K to 298 K to fully relax the model. Finally, the NVT ensemble was applied for 200 ps calculation at the required following simulation temperature to minimize the total energy of the models. The interface models after the above preprocessing were applied for further classical molecular dynamics simulation.

## 3. Results and Discussion

### 3.1. Macroscopic Fatigue Model Optimization

#### 3.1.1. Relationship Fitting Between Fatigue Life and Stress or Strain

**Figure 7:**
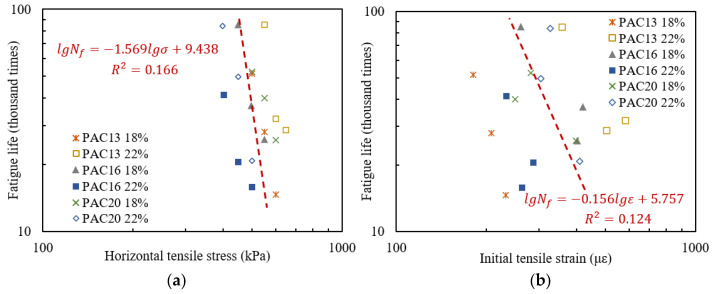
Correspondence between fatigue life and stress or strain of drainage asphalt mixture specimens: (**a**) horizontal tensile stress; (**b**) initial tensile strain.

From a macro perspective, the damage evolution law of drainage asphalt mixtures under cyclic loading is characterized by fatigue life. The fatigue life of various specimens in indirect tensile testing was tested, the corresponding tensile stress was recorded, and the initial tensile strain was calculated by Equation (2). The relationships between fatigue life and stress or strain are plotted in Figure 7.

The relationships between the fatigue life $N_{f}$ and the horizontal tensile stress σ or initial tensile strain ε of different types of specimens were fitted using exponential equations, as shown in Equation (5) [25]. By taking logarithms on both sides of Equation (5), a linear Equation (6) can be obtained. The relationships between the fatigue life and corresponding stress or strain of various types of specimens were fitted using the least squares method, respectively, and the results are shown in Table 5. The relationships between the fatigue life and the strain or stress of six types of specimens as a whole were further fitted and the results are marked in Figure 7.

(5) $$\left\{\begin{array}{c} N_{f}=a{\left(\frac{1}{\sigma }\right)}^{b}\\ N_{f}=c{\left(\frac{1}{\varepsilon }\right)}^{d} \end{array}\right.$$

(6) $$\left\{\begin{array}{c} lgN_{f}=-blg\sigma +lga\\ lgN_{f}=-dlg\varepsilon +lgc \end{array}\right.$$

As seen in Figure 7 and Table 5, the fatigue life–stress equation and fatigue life–strain equation have good fitting effects on drainage asphalt mixtures with the same void rate and gradation, with regression coefficients above 0.82. However, if the same exponential equation is used to fit the data of different types of specimens, the regression coefficient is less than 0.2, so there are significant differences in the fitting equations for mixtures with different void rates and gradations. The relationship between stress or strain and fatigue life is relatively superficial, with numerous influencing factors. Therefore, there is no fitting equation applicable to various types of drainage asphalt mixtures between fatigue life and horizontal tensile stress or initial tensile strain. It is necessary to establish corresponding relationships between other variables and fatigue life, in order to minimize the influence of material factors such as void rate and gradation.

#### 3.1.2. Fatigue Life Modeling Based on the Slope of Indirect Tensile Modulus Curve

**Figure 8:**
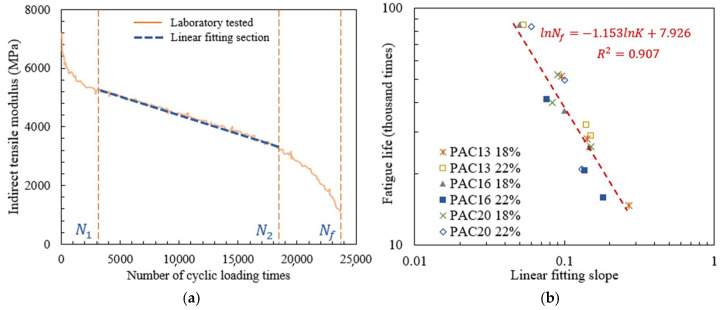
Fatigue life modeling based on indirect tensile modulus: (**a**) fitting of linear fitting slope; (**b**) relationship between fatigue life and linear fitting slope.

In indirect tensile testing, the modulus variation with the increase in the number of cyclical loading times was calculated by Equation (3), and the modulus attenuation curve was plotted. The modulus attenuation curve of the PAC-13 specimen with a void ratio of 18% in Figure 8a is taken as an example, and it is obvious that the curve can be divided into three stages from left to right. In current studies, exponential, linear, and exponential functions were used to fit the three stages, successively [7,8], but the method of dividing the boundaries of each stage was not clearly defined, resulting in the indeterminacy of the fitting results. In this paper, the following method was proposed to determine the linear fitting interval.

Firstly, the middle position of the linear stage was estimated, and the corresponding number of cyclic loading times was recorded as N. The interval of [N−100, N] was selected, and the least squares method was applied to fit the data inside the above interval, so as to obtain the regression coefficient $R^{2}$.

Secondly, the fitting interval was expanded to the right by 100 loading times, i.e., the [N−100, N+100] interval, and then the linear fitting was performed again to calculate the regression coefficient. Afterwards, the fitting interval was alternately expanded to the left and right successively, and the regression coefficients were continuously calculated. If the regression coefficients obtained after five consecutive left expansions decrease gradually, it indicates that the left side of the fitting interval has exceeded the linear stage. Then, the expanding of the fitting interval to the left was stopped, and the total number of left expansion times was recorded as $T_{l}$. Similarly, the total number of extensions to the right was denoted as $T_{r}$. Thus, the final linear fitting interval, i.e., the linear stage interval, was determined to be [$N-100\left(T_{l}-5\right),N+100\left(T_{r}-5\right)$].

Based on the indirect tensile modulus curves of the six different types of drainage asphalt mixture specimens, the linear fitting slope values K of the linear stage were calculated, and the results are shown in Table 6.

The relationship between the linear fitting slope K and fatigue life was described by an exponential formula, as shown in Equation (7) [7], and the logarithms were performed on both sides of the equation. The least squares method was applied to fit the data of each type of specimen and all of the six types of specimens, as shown in Figure 8b. The values of *p* and *q* were further calculated, as shown in Table 6 and Equation (8), respectively.

(7) $$\left\{\begin{array}{c} N_{f}=p{\left(\frac{1}{K}\right)}^{q}\\ lgN_{f}=-qlg\varepsilon +lgp \end{array}\right.$$

(8) $$N_{f}=2769.7{\left(\frac{1}{K}\right)}^{1.153}$$

As shown in Figure 8b, for all types of specimens, the regression coefficient between fatigue life and linear fitting slope reaches 0.921, indicating a significant correlation, and the regression results have good applicability for six types of drainage asphalt mixture specimens with different void ratios and gradations under different stress levels. In Table 7, the fitting results between fatigue life and the linear fitting slope of each type of specimens are close, with high correlation coefficients. Therefore, the fatigue life fitting method of matrix asphalt mixtures proposed in reference [7] is also applicable to drainage asphalt mixtures.

### 3.2. Damage and Failure Evolution Only Under Traffic Loading

#### 3.2.1. Under Different Number of Loading Times

The mesoscopic PAC13 models constructed in Section 2.3 were applied in the following simulations. The mesoscopic model without an interface was applied first. “Ductile damage initiation criterion” (DUCTCRT) index and “segment degradation” (SDEG) index are applied to quantify the results of the color map obtained from the mesoscopic simulation.

The aggregate was set to have no damage and failure, so the damage and failure only developed in the mortar. In the Ductile damage model, DUCTCRT index is a quantitative description of plastic accumulation in the mesostructure. The DUCTCRT value is between 0 and 1, and a value greater than 0 indicates that the element damage has begun to accumulate. When the value is equal to 1, it means that the cumulative damage of the element has reached its maximum. If the strain of the element continues to increase, failure begins to occur. The distribution diagram of DUCTCRT values for each element is called the damage distribution diagram. The “damage degree” indicator was proposed to characterize the overall damage situation of the mortar, and it is defined to be the average value of DUCTCRT weighted by the area of each mortar element at the time of loading completion.

In the Ductile damage model, SDEG index characterizes the stiffness of the element, with values ranging from 0 to 1. A result of 1 indicates that the element cannot withstand stress and has completely failed, so the crack generates, and the finite elements can be called the “cracked elements”. The distribution diagram of SDEG in each element is called the failure distribution diagram. The area weighted average value of SDEG of each high viscosity asphalt mortar element was defined to be the “failure degree” indicator, representing the overall failure situation of the mortar.

**Figure 9:**
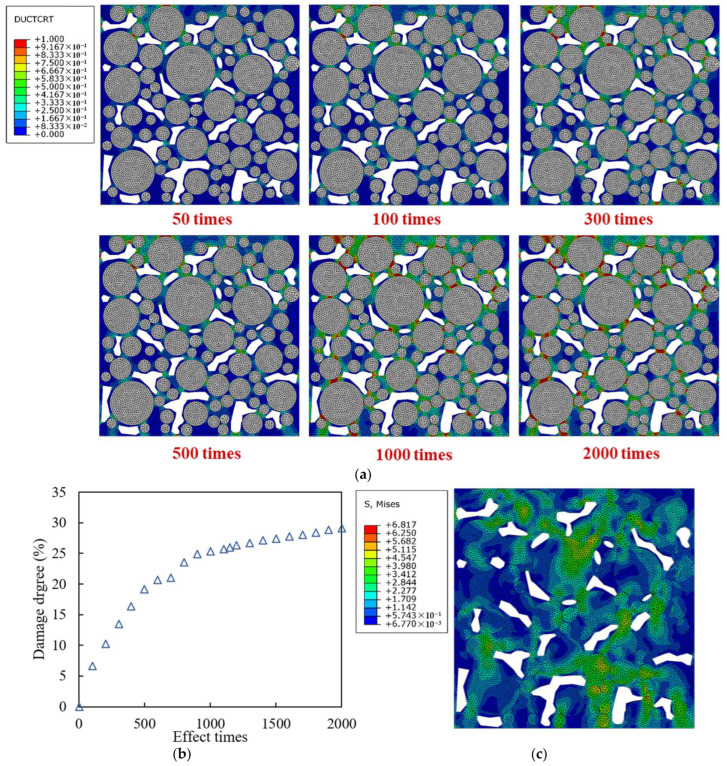
Damage and stress of models only under traffic loading with different effect times: (**a**) damage distribution diagrams; (**b**) damage degree development curve; (**c**) Mises stress distribution diagram under 2000 times of traffic loading.

Figure 9a shows the damage distribution of the mortar under different numbers of traffic loading time at 0.7 MPa and 10 Hz. The completely blank area is the void, the white grid area is the aggregate without damage, and the other areas are asphalt mortar. The damage degree curve under load is plotted in Figure 9b. Mises stress is the combination of tensile compressive stress and shear stress, which can reflect the internal stress distribution of the mortar, as shown in Figure 9c. It can be seen that the damage initially occurs in the area close to the load at the top of the model, and the area with better interlocking effect between aggregates has a higher stress level compared with other areas, thus transmitting the force chain. The mortar area on the force chain transmission path between adjacent aggregates is most severely damaged, while the damage in the mortar area outside the force chain is relatively small, and the damage development in the upper area of the model is faster than that in the middle and lower areas. When the load is applied for 500 times, the mortar damage accumulates rapidly, then the damage accumulation gradually slows down, and the damage degree eventually stabilizes at around 31%.

#### 3.2.2. Under Different Traffic Loading Levels

**Figure 10:**
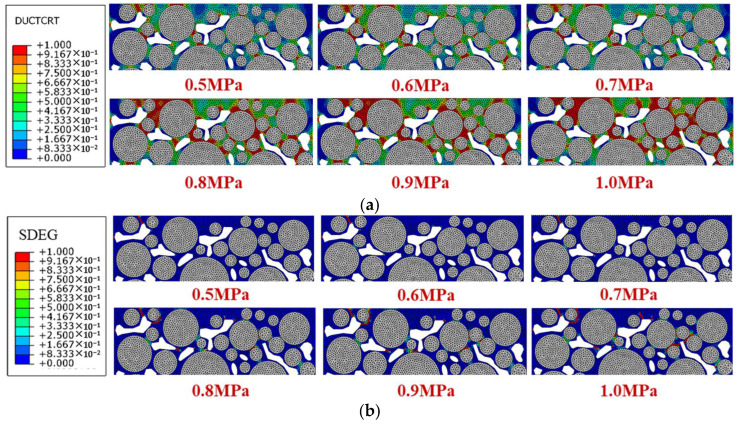
Damage and stress of models only under traffic loading with different loading levels: (**a**) damage distribution diagrams; (**b**) failure distribution diagrams.

Vehicle axle load and tire pressure influence the traffic load, so six traffic loading levels including 0.5 MPa, 0.6 MPa, 0.7 MPa, 0.8 MPa, 0.9 MPa, and 1.0 MPa were selected. The local damage and failure distribution after 2000 times of traffic loading is shown in Figure 10.

Figure 10a shows that with the increase in traffic loading, the damage area inside the high viscosity asphalt mortar spreads, and the red area where the damage accumulates to the maximum value increases. The damage increases significantly when the load increases from 0.7 MPa to 0.8 MPa, and when the load is less than 0.7 MPa or greater than 0.8 MPa, the mortar damage degree of each model is relatively close. It indicates that the increase in load has an uneven impact on the damage degree, and there is a critical load between 0.7 MPa and 0.8 MPa for the rapid development of damage.

The red area in Figure 10b is the crack, and the yellow and green areas represent the developing failure. When the traffic load is less than 0.7 MPa, there are fewer internal cracks and failure areas in the mortar. However, when the load is greater than 0.7 MPa, the damage develops rapidly with the increase in the load, and local cracks are interconnected to form macroscopic cracks. Cracks continue to increase and mainly appear around the aggregate, causing some of the aggregate to be stripped.

**Figure 11:**
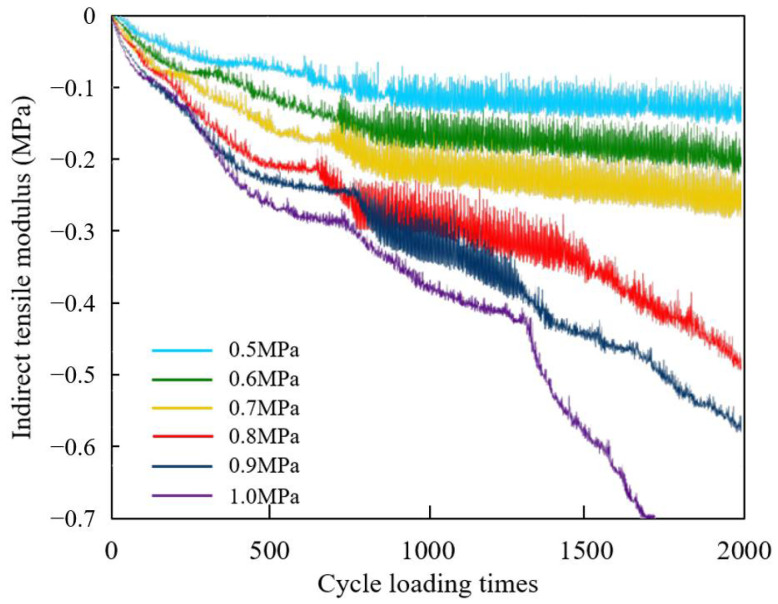
Displacement–time curves of indenter under different load levels.

The displacement–time curves of the indenter under different loads are shown in Figure 11. The larger the displacement of the indenter, the greater the deformation of the model. During a single traffic loading cycle, the displacement results are output once the load amplitude is zero and once the peak value is reached. Therefore, the displacement curve oscillates, and the magnitude of the oscillation reflects the rebound deformation of the model.

**Figure 12:**
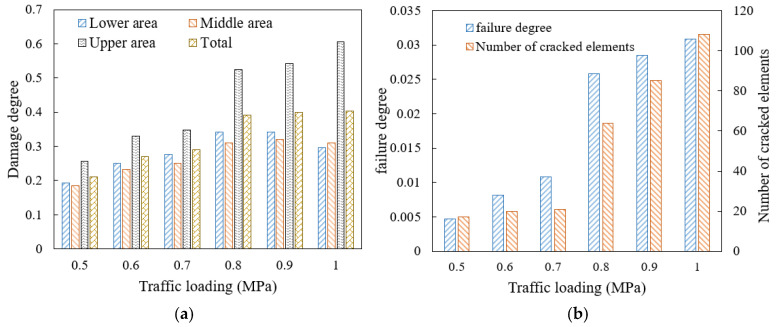
Damage and failure situation of mortar under different traffic loading levels: (**a**) damage degree; (**b**) failure degree and amount of cracked elements.

It shows in Figure 11 that a larger traffic load brings greater overall deformation of the model. When the load is below 0.7 MPa, it reaches a stable oscillation stage after about 700 loading times, while, when the load reaches 0.8 MPa or above, the deformation of the model rapidly increases after oscillation. When the load reaches 1.0 MPa, the model rapidly breaks down, so the oscillation region is not obvious. The higher the traffic load, the faster the later displacement development, and the model gradually transitions from plastic failure to brittle failure. Separating the model into the upper, middle, and lower areas from top to bottom averagely, the damage degree results of mortar under different traffic loads are shown in Figure 12a, and the failure degree and the amount of cracked elements are plotted in Figure 12b.

Figure 12 shows that when the traffic load increases from 0.7 MPa to 0.8 MPa, the increase rate of the damage and failure is the largest, while, under the load of 0.5 MPa and 0.6 MPa, the failure development is at a relatively low level. When the load increases from 0.5 MPa to 0.7 MPa, the number of cracked elements only increases by three, while, when the load increases from 0.7 MPa to 1.0 MPa, the above number increases by four times, indicating that heavy load has a significant impact on the damage and failure of mortar. If the SDEG value of an element reaches 1, the element cannot withstand stress, so it is automatically deleted. Consequently, when the load level reaches 1.0 MPa, the amount of the newly damaged element is less than the amount of the newly cracked elements due to the widespread crack in the mortar, reflected in the decrease in the damage degree and the rapid amount increase in the cracked elements. Therefore, restricting the over-loading vehicles passage is significant to slowing down the development of drainage pavement damage, considering that the 0.7 MPa loading is the conventional axle load.

Due to the direct contact load on the top of the model, the damage degree of the mortar in the upper area of the model is the most severe under different traffic loads. When the load increases from 0.5 MPa to 1.0 MPa, the damage degree of the mortar in the upper area increases by 100%, with the largest increase in the model. The increase rates of the damage degree of the mortar in the middle and the lower area are similar, and when the load is greater than 0.8 MPa, the damage degrees of the mortar in the middle and lower area increase slowly, while the mortar damage in the upper area is still rapidly developing. As the traffic load increases, the difference in mortar damage between the upper area of the model and other areas becomes larger. When the load reaches 1.0 MPa, the damage degree of the mortar in the upper area is twice as large than that of other areas.

### 3.3. Damage and Failure Under Coupling Effect of Traffic Loading and Hydrodynamic Pressure

#### 3.3.1. Evolution with and Without Hydrodynamic Pressure

**Figure 13:**
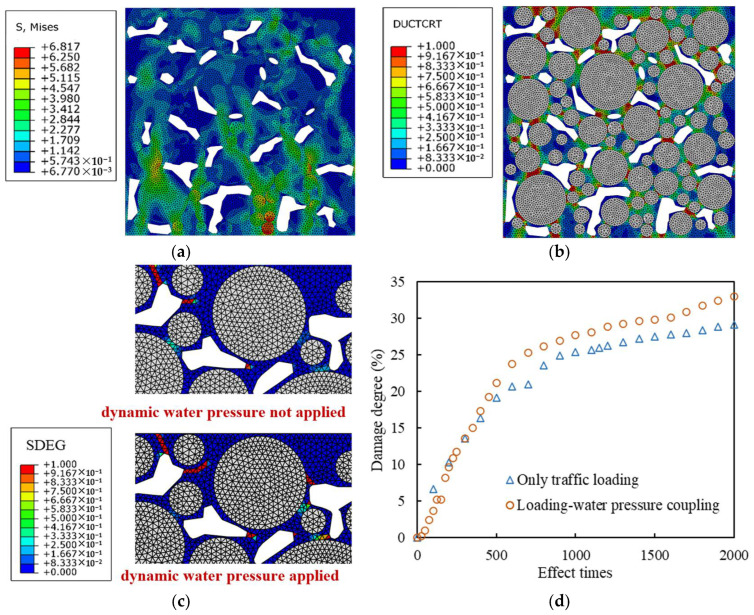
Stress, damage, and failure distribution diagrams and total damage development curves: (**a**) Mises stress distribution diagram; (**b**) damage distribution diagram; (**c**) failure distribution diagram; (**d**) total damage degree development curves of the mortar.

The traffic load and the hydrodynamic pressure were set to be 0.7 MPa and 0.2 MPa, respectively, with the operating frequency of 10 Hz. After 1500 times of coupling effect, the hydrodynamic pressure was canceled, and the traffic load continued to be applied 500 times. The evolution and final distribution of Mises stress, damage, and failure are shown in Figure 13.

Compared with when only traffic load is applied, the hydrodynamic pressure significantly increases the stress level of mortar around the voids and close to the aggregate, especially in the lower area of the model, promoting damage development and crack extension. The reason is that the hydrodynamic pressure exerts a squeezing effect on the mortar around the voids, and due to the fact that the stiffness of the aggregate is much greater than that of the mortar, the mortar experiences a greater reaction force at the interface with the aggregate.

The hydrodynamic pressure of 0.2 MPa does not significantly change the damage distribution area, but due to its stress effect on the mortar near the voids, new damage accumulation areas appear around some voids, and crack further develops along the crack tip in the failure area. Under the coupling effect of traffic loading and hydrodynamic pressure, the damage in the early stage is smaller, but after more than 300 coupling loading times, the damage develops more rapidly. When the hydrodynamic pressure is canceled after 1500 cyclical times, the damage development of the coupling loaded model is much faster than that of the model loaded by the traffic loading alone, which shows that hydrodynamic pressure reduces the load resistance of high viscosity asphalt mortar.

#### 3.3.2. Under Different Hydrodynamic Pressure Levels

**Figure 14:**
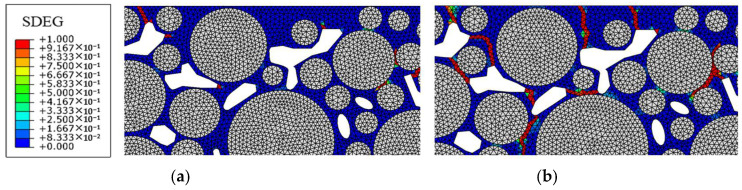
Failure distribution diagrams under different hydrodynamic pressure values: (**a**) 0.1 MPa; (**b**) 0.5 MPa.

The traffic load was taken as 0.7 MPa, and the hydrodynamic pressure was taken as five levels, i.e., 0.1 MPa, 0.2 MPa, 0.3 MPa, 0.4 MPa, and 0.5 MPa. Figure 14 shows the failure distribution diagrams when the hydrodynamic pressure is 0.1 MPa and 0.5 MPa, separately.

**Figure 15:**
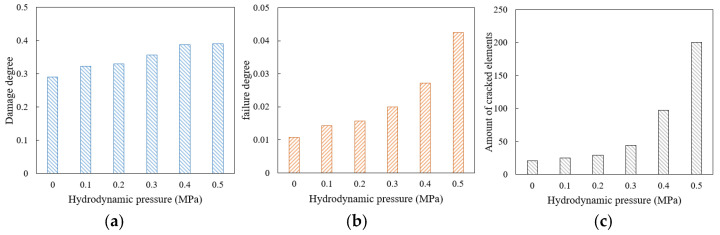
Damage and failure situation of mortar under different hydrodynamic pressure levels: (**a**) damage degree; (**b**) failure degree; (**c**) amount of cracked elements.

Compared with when only traffic load is applied, when the hydrodynamic pressure in the coupling effect is 0.3 MPa or below, only a few areas of the model show more obvious failure development, and new cracks appear in some weak mortar areas. When the hydrodynamic pressure reaches 0.5 MPa, more cracks mainly appear around the aggregate, indicating that the hydrodynamic pressure obviously destroys the adhesion between the aggregate and asphalt mortar. Cracks connect voids that are closer in distance, leading to more severe destruction to the macroscopic structure. The damage and failure results under different hydrodynamic pressure levels are shown in Figure 15.

It can be seen that the damage and failure degrees of the mortar increases with the increase in hydrodynamic pressure, and the failure and cracking show an accelerated growth trend. When the hydrodynamic pressure increases from 0.4 MPa to 0.5 MPa, the increase in damage degree is relatively slow, but it does not mean that the actual expansion rate of damage slows down. The reason is that at this time, the cracks develop rapidly, and the number of cracked elements increases by nearly double, resulting in the partial addition of mortar damaged elements being offset by the deletion of elements after cracking.

### 3.4. Damage and Failure of Mortar with Different Aging Degrees

**Figure 16:**
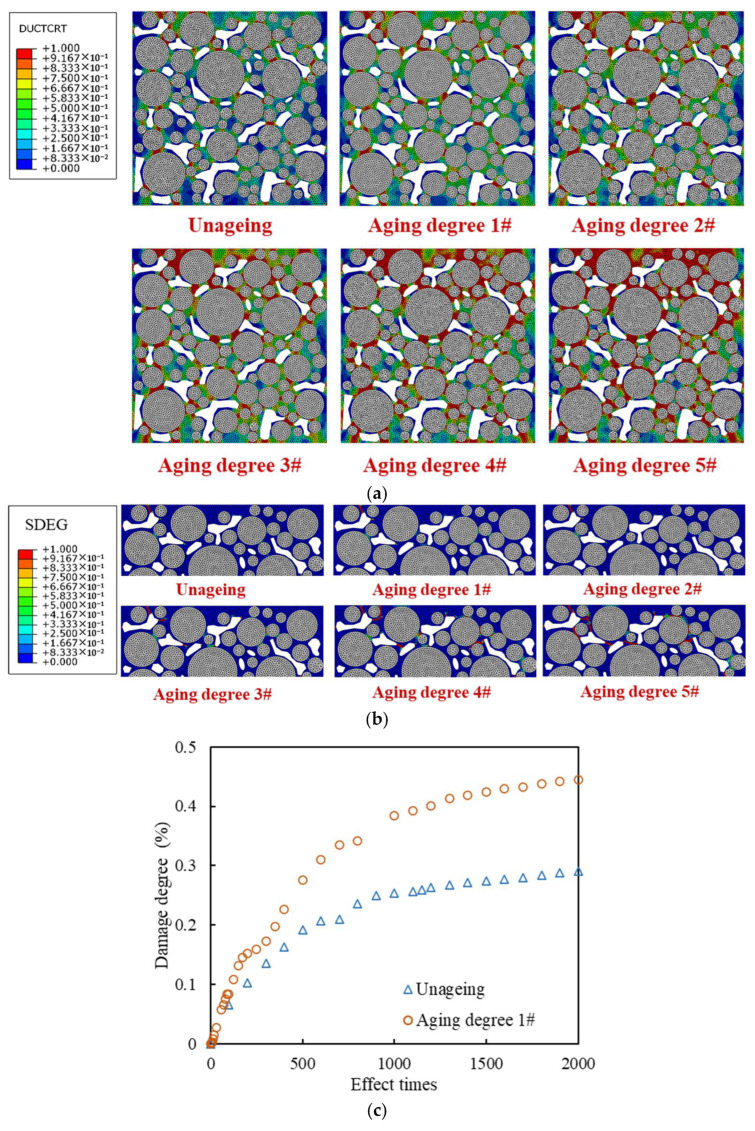
Damage and failure situation with different aging degrees: (**a**) damage distribution diagrams; (**b**) failure distribution diagrams; (**c**) total damage degree development curves.

According to the parameters including fracture strain and fracture energy obtained in Section 2.3, six sets of parameters were determined to characterize different aging stages of the high viscosity mortar. The 10 Hz, 0.7 MPa traffic load was applied on the top of the drainage asphalt mixture model with different mortar aging stages for 2000 cyclical times. The damage distribution diagram was output in Figure 16a, and the failure distribution diagram of the upper area of the model was output in Figure 16b. The total damage degree development curves of the unaged mortar and the mortar in aging degree 1# are plotted in Figure 16c for comparison.

**Figure 17:**
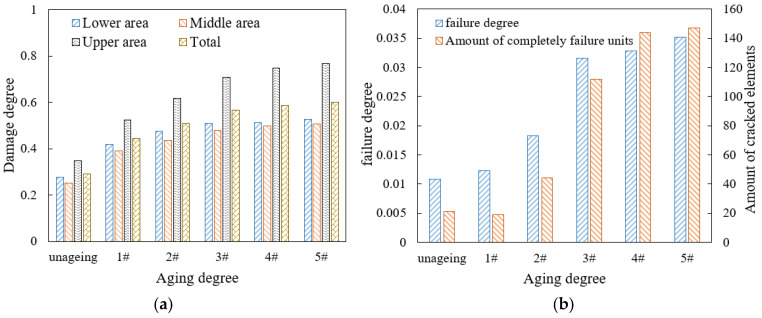
Damage and failure situation of mortar under different aging degrees: (**a**) damage degree; (**b**) failure degree and amount of cracked elements.

Due to the direct load on the top of the model, the damage in the upper area of the model with the mortar in aging degree 1# has reached 76.9%. As aging deepens, the damage area of mortar expands, and the element proportion with DUCTURT values exceeding 50% increases from 45.93% at aging degree 1# to 59.29% at aging degree 5#. In the upper area of the model, the number of the cracked elements around the aggregate significantly increases, and aging causes more aggregate to peel off. Compared with the unaged model, the damage development rate of the aged model increases. For the model with mortar in aging degree 1#, the damage degree after 500 cyclical times is similar to that of the unaged model after 2000 cyclical times, and its damage degree in the final stable stage increases by about 16 percentage points. The damage and failure situations of models with different aging degrees are shown in Figure 17.

It can be seen that the damage and failure degree of the mortar both increase with the deepening of aging. From unageing to aging degree 5#, the damage degree of mortar increases by 100%, with an increase of about 50% from the unageing stage to aging degree 1#, and then the increase rate gradually decreases. When the aging degree deepens from 2# to 3#, the failure degree increases by about 50% and the number of cracked elements increases by 68, accounting for 0.8% of the total mortar area, and then the increase rate of failure decreases when the aging further deepens.

The mortar damage in different areas inside the mixture model shows an upward trend with the deepening of aging degree. The upper area always has the highest damage degree, followed by the lower area and the middle area, and the increase rate of damage degree when aging deepens gradually decreases from top to bottom of the model. As aging deepens, damage accumulates most significantly in the upper area, and its proportion of total damage increases from 39.2% to 42.68% of the overall damage.

### 3.5. Damage and Failure of Mixture Considering the Interfacial Attenuation

#### 3.5.1. Parameter Representation of Interfacial Attenuation

The mesoscopic model considering the interface failure in Section 2.3 was applied, and the initial elastic modulus of the interface was set to be 3500 MPa, which is the same as that of the mortar. When damage occurs in the interface area, it can be characterized by the parameter decrease in elastic modulus, fracture strain, and fracture energy. Due to the fact that the damage of aggregate can be ignored, damage and failure develop simultaneously in the mortar and interface in the drainage asphalt mixture model. According to existing research, the range of fracture strain is about 0.01ε–0.03ε, and the range of fracture energy is about 2 kJ/m^2^–6 kJ/m^2^ [41,42,43]. Considering the above three parameters of interface elements, a three-factor three-level orthogonal experiment [44] was conducted, and variance analysis was conducted to obtain the significance levels of parameters. A higher significance level represents higher influence effect, and if the significance level is smaller than 0.05, the corresponding factor has obvious influence effect on the dependent variable. The results are shown in Table 8.

As shown in Table 7, the influence of interface parameters on the interface damage is significantly greater than that on mortar damage, but only fracture strain has a significant influence effect on interface damage results, followed by the fracture energy. The elastic modulus has little effect on the damage of both the interface and mortar; therefore, its value was uniformly selected as 3500 MPa in the following contents of this paper. When the interface is not considered to be an independent part, it can be regarded that the interface has the same plastic parameters as same as those of the unaged mortar, and it can also be seen as the interface state without attenuation. The parameter values for different interfacial failure degrees were determined on this basis, as shown in Table 9.

For the models with dependent interfaces between the aggregate and mortar, the overall damage degree ${DUCTCRT}_{total}$ and failure degree ${SDEG}_{total}$ were weighted by the area of interface and mortar elements, as shown in Equations (9) and (10). In the equations, N and M are the total amount of mortar elements and interface elements, respectively. ${DUCTCRT}_{mortar,i}$ and ${DUCTCRT}_{interface,j}$ are the DUCTCRT values of the ith(1≤i≤N) mortar element and the jth(1≤j≤M) interface element, respectively. $S_{mortar,i}$ and $S_{interface,j}$ are the areas of the ith(1≤i≤N) mortar element and the jth(1≤j≤M) interface element, respectively.

(9) $${DUCTCRT}_{total}=\frac{\sum_{i=1}^{N}S_{mortar,i}\times {DUCTCRT}_{mortar,i}+\sum_{j=1}^{M}S_{interface,j}\times {DUCTCRT}_{interface,j}}{\sum_{i=1}^{N}S_{mortar,i}+\sum_{j=1}^{M}S_{interface,j}}$$

(10) $${SDEG}_{total}=\frac{\sum_{i=1}^{N}S_{mortar,i}\times {SDEG}_{mortar,i}+\sum_{j=1}^{M}S_{interface,j}\times {SDEG}_{interface,j}}{\sum_{i=1}^{N}S_{mortar,i}+\sum_{j=1}^{M}S_{interface,j}}$$

#### 3.5.2. Different Interfacial Attenuation Degrees

**Figure 18:**
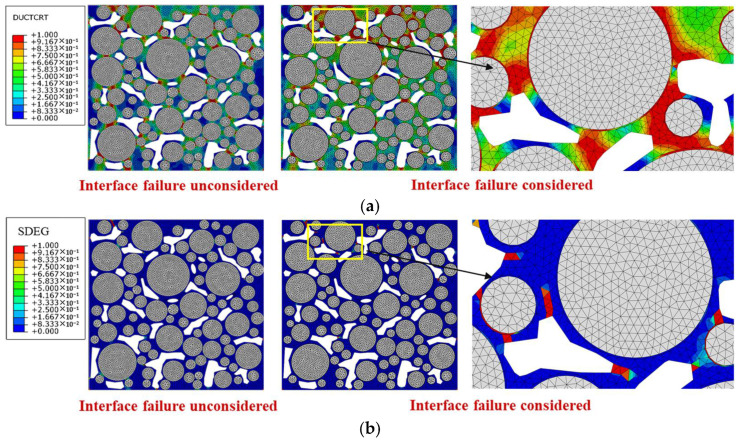
Damage and failure distribution after 2000 times of traffic loading: (**a**) damage distribution diagrams; (**b**) failure distribution diagrams.

The interfacial attenuation degree 3# was taken as an example, and 2000 cyclical times of the 0.7 MPa and 10 Hz traffic loading was applied. The final results of the damage and failure distribution are shown in Figure 18.

It can be seen that when the interfacial attenuation degree is 1#, compared with the state without interfacial attenuation, the damage area and failure area of mortar increase by 10.9% and 6.8%, respectively, and the damage degree and failure degree of mortar increase by 59.6% and 24.6%, respectively. The main reason is that the thin interface has a weak ability to withstand plastic strain, and it undergoes large plastic strain under load, resulting in stress concentration in the surrounding mortar area. Compared with the surrounding mortar, the interface damage is significantly more severe, indicating that the interface is a weak area of the drainage asphalt mixture under load. Considering the interfacial attenuation, new crack development areas appear at the boundary tips of some voids in the model, leading to the aggregate stripping from the mortar.

**Figure 19:**
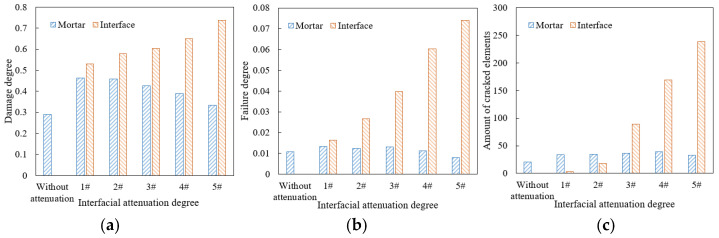
Damage and failure situation of mortar and interface: (**a**) damage degree; (**b**) failure degree; (**c**) amount of cracked elements.

Based on the parameters in Table 8, the models with different interfacial attenuation degrees were constructed, and the traffic loading was set as 0.7 MPa and 10 Hz for 2000 cyclical times. The damage and failure results of mortar and interface elements are shown in Figure 19.

**Figure 20:**
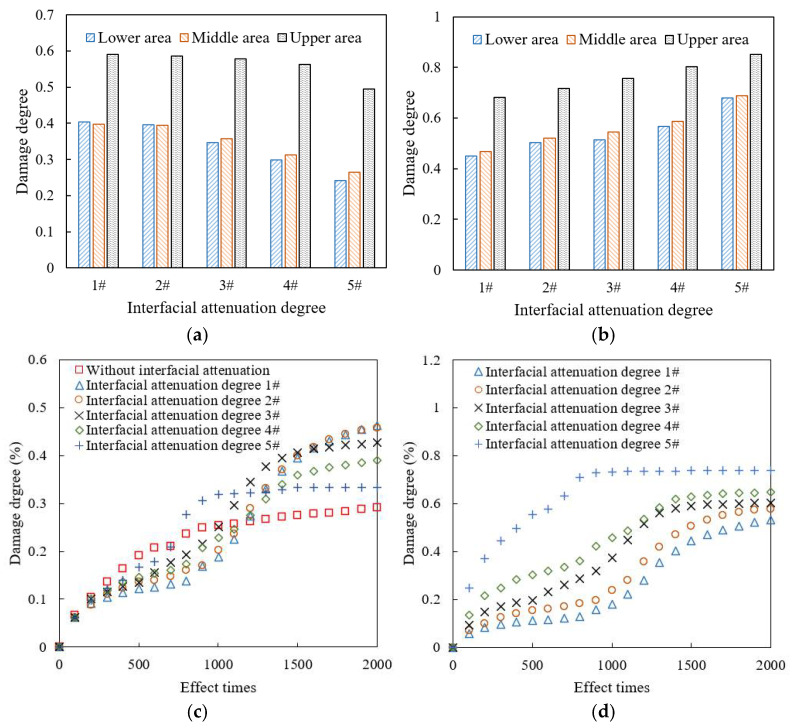
Damage degree under different interfacial attenuation degrees: (**a**) mortar, final damage degree; (**b**) interface, final damage degree; (**c**) mortar, damage degree increase curve; (**d**) interface, damage degree increase curve.

As shown in Figure 19, as the interfacial attenuation degree deepens from 1# to 5#, the damage degree of mortar decreases by about 28.0%, while that of interface increases by about 38.9%. The failure degree of mortar increases by 41.4%, while that of interface increases by 350%. The number of cracked elements in the mortar remains relatively stable, but that in the interface increases by nearly a hundred times. Accordingly, after the interfacial attenuation develops to a certain extent, the interface damage and failure develop rapidly, but the damage and failure of the mortar weaken. The reason is that as the interfacial attenuation deepens, the plasticity of the interface is significantly lower than that of the surrounding mortar, and the load transfer ability from the interface to the mortar decreases. The load stress is more concentrated in the interface area, which limits the transmission of the internal force chain. Although the damage and failure of mortar are temporarily reduced, as the number of loading times increases, when the interface completely cracks, the aggregate will be quickly stripped, causing rapid damage to the mixture and seriously affecting the integrity of the drainage asphalt pavement. Therefore, the above-mentioned damage degree and failure degree reduction in mortar is unfavorable for the long-term load resistance of the mixture. The damage degree values in the upper, middle, and lower areas of the model are plotted in Figure 20.

As shown in Figure 20a,b, under different interfacial attenuation degrees, the damage degree of mortar and interface in the upper area of the mixture model is significantly higher than that of the middle and lower areas, and the damage degree of the interface is always much higher than that of the mortar. As the interfacial attenuation deepens, the damage degree of the interface increases, while the damage degree of the mortar decreases. The proportion of mortar damage of the upper area in the overall mortar damage gradually increases. When the interfacial attenuation degree reaches 5#, the mortar damage of the upper area is close to 50% of the overall mortar damage of the model.

As shown in Figure 20c,d, with the increase in load cyclical times, when the interface is not attenuated, that is, its plastic parameters are consistent with the surrounding mortar, the increase in mortar damage continues to slow down. However, when the interface attenuates, i.e., its plasticity is weaker than that of the surrounding mortar, the damage degree of the mortar develops in multiple stages. When the traffic load is within 200 loading times, the damage develops rapidly, and then the damage development gradually slows down. After 800 loading times, the damage development continues to accelerate, while it gradually slows down after 1500 loading times. The reason is that damage accumulates rapidly at the mortar in the strong force chain during the initial loading stage. After that, the interface damage and failure become more severe and the force chain transmitting is limited, which makes the loading force to be more concentrated in the interface areas. The damage accumulates rapidly again after the force chain is redistributed, until the model is obviously damaged and cracked. Consequently, the more severe the interface attenuation, the faster the mortar damage develops to the final stable stage.

The more severe the interface attenuation, the higher the interface damage degree under the same number of traffic loading times. When the interfacial attenuation degree is better than 4#, the evolution curve of interface damage degree can also be divided into multiple stages, namely, slow growth within 1000 loading times, rapid growth between 1000 and 1500 loading times, and final stabilization after 2000 loading times. The more severe the interface attenuation, the earlier the interface damage degree reaches the stable stage. When the interfacial attenuation level reaches 5#, the interface damage degree reaches a stable stage after about 800 loading times. The reason is that at this time, the model is severely damaged, with a large amount of aggregate stripping and local mortar fracture, making it difficult for the subsequent applied loads to be effectively transmitted and spread in the model. Therefore, ensuring the adhesion characteristics and mechanical properties of the interface is the key to extending the service life of the drainage asphalt mixture.

### 3.6. Damage and Failure Under Coupling Effect of Multiple Loading and Material Factors

#### 3.6.1. Orthogonal Experiment Setting

The multifactor coupling effect of traffic loading, hydrodynamic pressure, mortar aging, and interfacial attenuation were further considered. The traffic loading was set at four levels of 0.5 MPa, 0.7 MPa, 0.8 MPa, and 0.9 MPa, and the hydrodynamic pressure was set at four levels of 0.1 MPa, 0.2 MPa, 0.3 MPa, and 0.4 MPa. The aging degree and interfacial attenuation degree levels of 1# to 4# in Table 6 and Table 8 were selected, respectively. The traffic loading and hydrodynamic pressure were both applied for 2000 times with the frequency of 10 Hz. The factor combinations in the four-factor four-level orthogonal experiment [45,46] were determined, as seen in Table 10.

#### 3.6.2. Significance Levels of Factors and Macroscopic Fracture Forms

**Figure 21:**
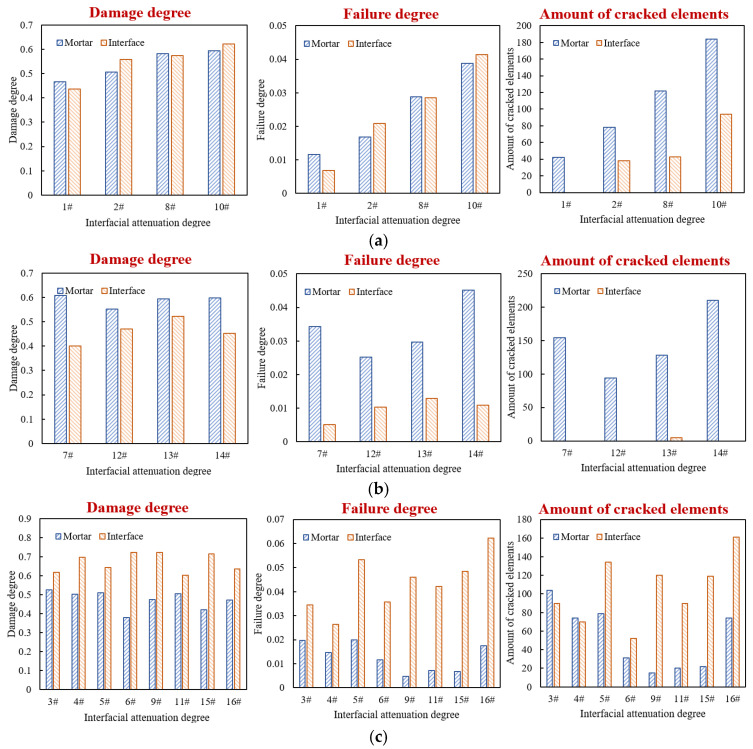
Damage degree, failure degree, and number of cracked elements of various models: (**a**) Group 1#; (**b**) Group 2#; (**c**) Group 3#.

The damage degree, failure degree, and number of cracked elements of mortar and interface under each combination were calculated, as shown in Figure 21.

**Figure 22:**
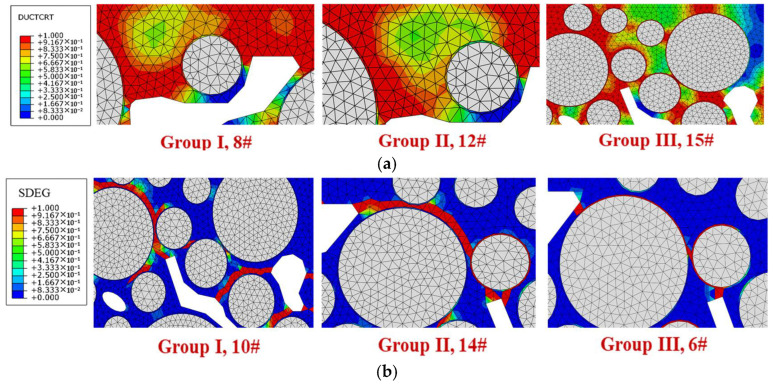
Damage and failure area distributions under different multifactor combinations: (**a**) damage distribution diagrams; (**b**) failure distribution diagrams.

Accordingly, the 16 factor combinations can be divided into three groups. Group I includes combinations 1#, 2#, 8#, and 10#; the differences in the damage degree of interface and mortar are within 5%, corresponding to the general adhesion state between asphalt and aggregate in actual drainage pavement. Group II includes combinations 7#, 12#, 13#, and 14#; the interface damage degree is lower than that of mortar, and the differences are greater than 7%, corresponding to the good adhesion state between asphalt and aggregate in actual drainage pavement. In Group III, the interface damage degree is greater than that of mortar, corresponding to situations where the adhesion between asphalt and aggregate is poor or the interface water damage is severe in actual drainage pavement. For combinations 3#, 4#, 5#, and 11#, the damage degree differences are less than 20%, while for combinations 6#, 9#, 15#, and 16#, the differences are more than 20%. The damage and failure distribution of each model are shown in Figure 22.

The variance analysis was conducted to calculate the significance levels of each factor, as shown in Table 11. If the significance level is less than 0.05, it indicates that the factor has a significant impact on the corresponding dependent variable, and a lower significance level represents a higher influence effect.

It can be seen that for the three indicators of the damage degree, failure degree, and number of cracked elements of both mortar and interface, the impacts of interfacial attenuation and mortar aging factors are always in the first or second place, while the impacts of traffic loading and hydrodynamic pressure are always in the third or fourth place. For interface, interfacial attenuation has a much greater impact on the three above indicators than the other factors, indicating that the plasticity of the interface is the fundamental influencing factor for its development of damage and failure.

As for the three above indicators of mortar, the aging degree of mortar is the most influential factor, and the influence of interfacial attenuation degree is slightly lower. The hydrodynamic pressure promotes the damage and failure of the mortar, but it has a relatively small impact on the development of interface damage and failure. The reason is that the asphalt film of the drainage asphalt mixture is thick, and the mortar can better wrap the aggregate. The interface elements between aggregate and mortar are less affected by the hydrodynamic pressure acting in voids.

For Group I, as shown in Figure 20a, when the damage degree values of mortar and interface are close, their failure degree values are also close. The number of cracked mortar elements is greater than that of interface elements but considering the huge difference between the element numbers of mortar and interface, the cracked element proportion of interface is much higher than that of the mortar. As shown in Figure 21, cracks appear simultaneously in both the mortar and interface, so there is stripping of individual aggregates and aggregates wrapped in mortar. Due to the significant hydrodynamic pressure, there are many cracks developing from the tip of the void boundary inside the model, with some cracks along the interface and others along the weak areas of the mortar.

For Group II, as shown in Figure 20b, when the damage degree of mortar is greater than that of the interface, the failure degree of mortar is also greater. Due to the low interfacial attenuation degree, the failure degree is also low, and there are almost no cracked elements on the interface. As shown in Figure 21, under high hydrodynamic pressure, a large number of cracks are formed along the tip of the void boundary. The cracks mainly extend around the aggregate, passing through the weak areas of mortar, connecting with other nearby voids, and forming macroscopic cracks. It leads to the stripping of the aggregate wrapped in mortar, especially in the upper area of the model.

For Group III, as shown in Figure 20c, when the damage degree of interface is greater than that of mortar, the failure degree of interface is also greater than that of mortar. The interface attenuation degree of each model in this group is more severe than or close to the aging degree of mortar. At this time, the interface has greater strain, while the deformation of the mortar is smaller, so the interface is prone to cracking. The damage accumulation of nearby mortar is also slowed down due to the obstruction of stress transmission, so the damage degree of interface is higher than that of the mortar. As shown in Figure 21, cracks develop at the tip of the void boundary propagate along the interface, while the nearby mortar has a lower failure degree, so the destruction of the drainage asphalt mixture is mainly manifested as aggregate stripping without mortar.

### 3.7. Microscopic Molecular Influence of Water on Interfacial Adhesion

In the above studies, hydrodynamic pressure was only applied in the voids, but when serious destruction occurs in the drainage asphalt mixture, water enters the interface between the aggregate and mortar through cracks in mortar, exacerbating the attenuation of interfacial adhesion. Interface adhesion is formed by the interaction between aggregates and high viscosity asphalt in the mortar. Based on molecular dynamics simulation, the hydrogen bond distribution on the interface in water containing conditions was analyzed. The interaction energy of the interface in non-water-containing or water-containing environments were compared, so as to clarify the molecular mechanism of this process. The interface models with and without water in Figure 6 were applied, and a 1 ns NPT (i.e., the number of atoms N, pressure P, and temperature T in the model remained constant) ensemble simulation was performed at 25 °C and 1 atm with a time step of 1 fs.

Hydrogen bonds affect intermolecular interactions, and there are six types of hydrogen bonds at interfaces in water environments, namely, hydrogen bonds between asphalt and asphalt, water and asphalt, aggregate and asphalt, aggregate and aggregate, aggregate and water, water and water [47]. The proportion of four types of hydrogen bonds related to interface adhesion was calculated, including water–asphalt, water–aggregate, water–water, and aggregate–asphalt hydrogen bonds.

**Figure 23:**
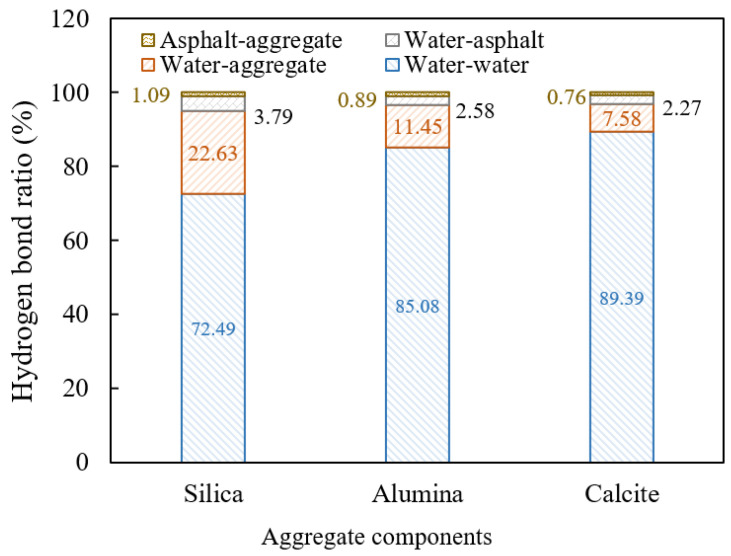
Proportions of different hydrogen bonds at the interface.

As shown in Figure 23, water–water hydrogen bonds account for the vast majority of the total interface hydrogen bonds in a water containing environment, followed by water–aggregate and water–asphalt hydrogen bonds, and the asphalt–aggregate hydrogen bonds only account for about 1%. It indicates that the aggregate significantly tends to form hydrogen bonds with water rather than asphalt, thus attenuating the interfacial adhesion. For the interface between the asphalt and aggregate, the asphalt–aggregate hydrogen bonds are conducive to the adhesion of the interface, while the water–aggregate and water–asphalt hydrogen bonds promote the stripping of the interface. In this regard, the “interface hydrogen bond ratio (IHR)” indicator was proposed, which is defined as the ratio of the sum of water–aggregate and water–asphalt hydrogen bonds to the asphalt–aggregate hydrogen bonds. The greater the HIR value, the greater the promotion effect of hydrogen bond on interface stripping. According to the data in Figure 23, the IHR indicators of the interface between high viscosity asphalt and silica, alumina, and calcite aggregate components were calculated to be 24.24, 15.76, and 9.83, respectively. It can be seen that the IHR indicator between high viscosity asphalt and silica is much higher than that with alumina or calcite aggregate components, indicating that silica, as an acidic aggregate component, has obvious hydrophilicity and is not conducive to the adhesion of high viscosity asphalt.

**Figure 24:**
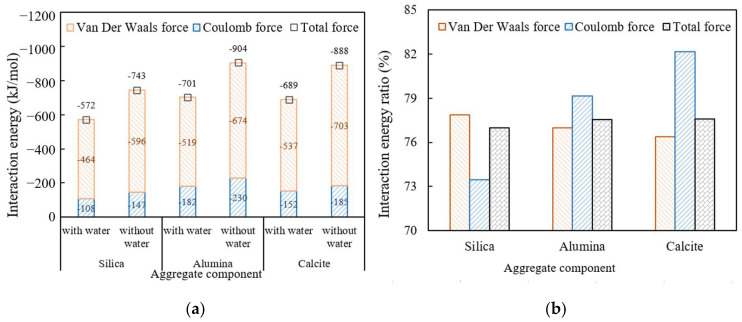
Absolute values of the interaction energy between high viscosity asphalt and different aggregate components: (**a**) interaction energy; (**b**) interaction energy ratio.

The interaction energy values between high viscosity asphalt and three aggregate components, namely silica, alumina, and calcite were calculated. Due to the mutual attraction between asphalt and aggregate, their interaction energy values are all negative, as shown in Figure 24a. The larger the absolute value of the interaction energy, the better the adhesion state between high viscosity asphalt and aggregate. The ratios of the interaction energy values in water-containing and non-water-containing conditions are shown in Figure 24b. The smaller the ratio, the poorer the water damage resistance of interface.

It can be seen that with the addition of water in the interface, the absolute values of van der Waals and Coulomb interaction energy between high viscosity asphalt and various aggregate components decrease, indicating that moisture reduces the interfacial adhesion. No matter in water-containing or non-water-containing conditions, the absolute value of the interaction energy between high viscosity asphalt and alumina or calcite is significantly greater than that of silica, indicating that the acidity and alkalinity of aggregates still have a significant impact on interfacial adhesion under water containing conditions. The ratio of interaction energy between high viscosity asphalt and silica is smaller than that between high viscosity asphalt and alumina and calcite, indicating that the water resistance of the interface with an acidic aggregate component is obviously lower than that the interface with alkaline aggregate components. Therefore, the alkaline aggregate is strongly applied in drainage asphalt mixtures. The van del Waals interaction energy is always the main interaction of interface adhesion in drainage asphalt mixtures at the molecular microscopic level.

## 4. Conclusions

In this paper, macroscopic fatigue tests, mesoscopic finite element simulations, and microscopic molecular dynamics simulations were composed to study the damage and failure of drainage asphalt mixtures in multiscale. The conclusions are as follows:

(1) There are good fitting effects between the fatigue life and the horizontal tensile stress or initial tensile strain for drainage asphalt mixtures with the same void rate and gradation, but there is no fitting equation applicable to various types of drainage asphalt mixtures. In view of this, a fitting method to calculate the linear fitting slope of the indirect tensile modulus curves was proposed, and the fatigue model based on the linear fitting slope has significant correlation for drainage asphalt mixtures with different void rates and gradations.
(2) The damage and failure have an obvious leap increase when the traffic loading increases from 0.7 MPa to 0.8 MPa. The mortar area on the force chain transmission path between adjacent aggregates is most severely damaged. The upper area always has the highest damage degree, followed by the lower and the middle area. The damage and failure and their development rates in the upper area are faster than that in the middle and lower areas. The hydrodynamic pressure significantly increases the stress level of mortar around the voids and close to the aggregate, especially in the lower area, promoting damage development and crack extension. When the hydrodynamic pressure in the coupling effect is greater than 0.3 MPa, the increase rates of damage and failure accelerate. Therefore, restricting the over-loading vehicles passage is significant to slowing down the development of drainage pavement damage, considering that the 0.7 MPa loading is the conventional axle load and the excessive traffic loading increases the hydrodynamic pressure obviously.
(3) With the development of interfacial attenuation, the damage and failure of interface continue increasing, while that of the mortar increases first and then decreases, which is related to the loading concentration in the interface and the decrease in stress level in the mortar. Therefore, ensuring the adhesion characteristics and mechanical properties of the interface is the key to extending the service life of the drainage asphalt mixture. When the interfacial plastic parameters are consistent with the surrounding mortar, the damage increase in mortar continues to slow down during traffic loading. However, when the interface attenuates and its plasticity is weaker than that of the surrounding mortar, the damage degrees of mortar or interface develop in multiple stages, which can be explained by the force chain limitation and redistribution by the interface destruction.
(4) The impact of interfacial attenuation and mortar aging factors on the damage and failure of the mortar or interface is always in the first or second place, followed by the traffic loading and hydrodynamic pressure factors. When the damage degree values of the mortar and the interface are close, cracks appear simultaneously in both the mortar and interface. When the damage degree of mortar is greater than that of the interface, cracks mainly extend around the aggregate, leading to the stripping of the aggregate wrapped in mortar. Conversely, cracks that develop at the tip of the void boundary propagate along the interface, while the nearby mortar has a lower failure degree, which is corresponding to the aggregate stripping without mortar.
(5) The van del Waals force is the main molecular effect of interface adhesion in drainage asphalt mixtures. The proportion of water–aggregate hydrogen bonds is significantly higher than that of the asphalt–aggregate hydrogen bonds, indicating that the aggregate significantly tends to form hydrogen bonds with water rather than asphalt, thus attenuating the interfacial adhesion. According to the hydrogen bond and interaction energy results, acidic aggregate component silica shows obvious hydrophilicity than alkaline aggregate components including alumina and calcite according to the hydrogen bond proportion, which is not conducive to the adhesion of high viscosity asphalt. Therefore, the alkaline aggregate is strongly applied in drainage asphalt mixtures.

## Figures and Tables

**Table 1:** Indicators of high viscosity asphalt, aggregate, and mineral powder.

Indicators	Asphalt	Coarse Aggregate	Fine Aggregate	Mineral Powder
Penetration (0.1 mm)	44.6	/	/	/
Softening point (°C)	91.3	/	/	/
5 °C ductility (cm)	52.4	/	/	/
60 °C viscosity (Pa·s)	152,650	/	/	/
Crushing value (%)	/	7.8	/	/
Los Angeles abrasion loss (%)	/	7.5	/	/
Needle flake content (%)	/	5.3	/	/
Sand equivalent (%)	/	/	76.18	/
Apparent density (g/cm^3^)	/	2.942	2.955	2.688
Bulk volume density (g/cm^3^)	/	2.875	2.890	2.688

**Table 2:** Gradation of different drainage asphalt mixtures.

Sieve Sizes (mm)		26.5	19	16	13.2	9.5	4.5	2.36	1.18	0.6	0.3	0.15	0.075
Passing rate (%)	PAC-13	100	100	100	96	54	18	11	10	8	6.5	4.5	4
	PAC-16	100	100	95	75	50	18	14.5	12	10	8	6.5	4
	PAC-20	100	97.2	90	80	64	26	17	14	10.5	8.5	5.8	4.8

**Table 3:** Loading stress levels of different specimens in indirect tensile testing.

Stress Level (kPa)	Void Rate 18%			Void Rate 22%		
	PAC13	PAC16	PAC20	PAC13	PAC16	PAC20
400	√			√		
450	√			√	√	
500	√	√		√	√	√
550		√	√		√	√
600		√	√			√
650			√			

**Table 4:** Parameter settings of mortar in different aging degrees.

Aging Degree	Unaged	1#	2#	3#	4#	5#
Fracture strain	0.343	0.03	0.025	0.02	0.015	0.01
Fracture energy (kJ/m^2^)	5.97	5	4.5	3.5	2.5	2

**Table 5:** Fitting results of the relationship between fatigue life and stress or strain.

Parameters	18%PAC13	22%PAC13	18%PAC16	22%PAC16	18%PAC20	22%PAC20
a	1.059 × 10^10^	5.56 × 10^16^	1.04 × 10^15^	1.06 × 10^9^	1.61 × 10^10^	3.31 × 10^14^
b	0.0370	0.0562	0.0593	0.0285	0.0304	0.0629
c	8.18 × 10^7^	2.36 × 10^7^	3.59 × 10^7^	1.31 × 10^6^	2.14 × 10^6^	2.03 × 10^8^
d	0.0728	0.0251	0.0348	0.0437	0.0332	0.0403
$R^{2}$, strain	0.868	0.952	0.823	0.915	0.924	0.944
$R^{2}$, stress	0.906	0.963	0.884	0.897	0.959	0.910

**Table 6:** Linear fitting slope values of six different types of drainage asphalt mixture specimens.

Stress Levels (kPa)	Void Rate 18%			Void Rate 22%		
	PAC13	PAC16	PAC20	PAC13	PAC16	PAC20
400	0.095			0.083		
450	0.142			0.090	0.053	
500	0.270	0.076		0.156	0.141	0.065
550		0.135	0.051		0.153	0.107
600		0.182	0.103			0.130
650			0.145			

**Table 7:** Fitting results of the relationship between fatigue life and linear fitting slope.

Parameters	18%PAC13	22%PAC13	18%PAC16	22%PAC16	18%PAC20	22%PAC20
p	2438.89	2540.71	2841.82	2232.55	2883.90	2123.24
q	1.1905	1.0249	1.1304	1.1277	0.9606	1.0921
$R^{2}$	0.988	0.969	0.957	0.946	0.925	0.902

**Table 8:** Orthogonal experimental results of interfacial attenuation parameters.

Orthogonal Factor Combinations and Significance Levels		Elastic Modulus (MPa)	Fracture Strain	Fracture Energy (kJ/m^2^)	Damage Degree of Mortar	Damage Degree of Interface
Combinations	1#	3500	0.03	6	0.45	0.53
	2#	2500	0.02	6	0.47	0.66
	3#	1500	0.01	6	0.41	0.77
	4#	1500	0.02	4	0.40	0.57
	5#	2500	0.03	4	0.43	0.48
	6#	3500	0.01	4	0.41	0.80
	7#	3500	0.02	2	0.32	0.47
	8#	2500	0.01	2	0.33	0.74
	9#	1500	0.03	2	0.42	0.44
Significance levels	For damage degree of mortar	0.840	0.525	0.250	/	/
	For damage degree of interface	0.766	0.034	0.198	/	/

**Table 9:** Parameter values of different interfacial attenuation degrees.

Interfacial Attenuation Degrees	Without Attenuation	1#	2#	3#	4#	5#
Fracture strain	0.343	0.03	0.025	0.02	0.015	0.01
Fracture energy (kJ/m^2^)	5.97	5	4	3	5	1

**Table 10:** Coupling multifactor combinations in orthogonal experiments.

Combinations	Traffic Loading (MPa)	Hydrodynamic Pressure (MPa)	Aging Degrees	Interfacial Attenuation Degrees
1#	0.5	0.1	1	1
2#	0.5	0.2	2	2
3#	0.5	0.3	3	3
4#	0.5	0.4	4	4
5#	0.7	0.1	2	3
6#	0.7	0.2	1	4
7#	0.7	0.3	4	1
8#	0.7	0.4	3	2
9#	0.8	0.1	3	4
10#	0.8	0.2	4	3
11#	0.8	0.3	1	2
12#	0.8	0.4	2	1
13#	0.9	0.1	4	2
14#	0.9	0.2	3	1
15#	0.9	0.3	2	4
16#	0.9	0.4	1	3

**Table 11:** The significance levels of various factors on different dependent variables.

Factors	Damage Degree		Failure Degree		Number of Cracked Elements	
	Mortar	Interface	Mortar	Interface	Mortar	Interface
Traffic loading	0.166	0.297	0.111	0.177	0.153	0.258
Hydrodynamic pressure	0.503	0.705	0.087	0.891	0.047	0.769
Aging degrees	0.004	0.120	0.015	0.103	0.009	0.093
Interfacial attenuation degrees	0.004	0.001	0.011	0.008	0.010	0.004

## Data Availability

The original contributions presented in this study are included in the article. Further inquiries can be directed to the corresponding author.

## Abbreviations

The following abbreviations are used in this manuscript:

CT	Computed Tomography
SBS	Styrene–Butadiene–Styrene
